# Worldwide Presence of National Anesthesia Societies on Four Major Social Networks in 2021: Observational Case Study

**DOI:** 10.2196/34549

**Published:** 2022-07-20

**Authors:** Thomas Clavier, Emilie Occhiali, Claire Guenet, Naurine Vannier, Camille Hache, Vincent Compere, Jean Selim, Emmanuel Besnier

**Affiliations:** 1 Department of Anesthesiology, Critical Care and Perioperative Medicine Rouen University Hospital Rouen France; 2 Rouen Medical School University of Rouen Normandy Rouen France

**Keywords:** social network, social media, anaesthesia, society, Facebook, Twitter, Instagram, YouTube

## Abstract

**Background:**

Although the presence of medical societies on social networks (SNs) could be interesting for disseminating professional information, there is no study investigating their presence on SNs.

**Objective:**

The aim of this viewpoint is to describe the worldwide presence and activity of national anesthesia societies on SNs.

**Methods:**

This observational study assessed the active presence (≥1 post in the year preceding the collection date) of the World Federation of Societies of Anesthesiologists member societies on the SNs Twitter, Facebook, Instagram, and YouTube. We collected data concerning each anesthesia society on the World Federation of Societies of Anesthesiologists website.

**Results:**

Among the 136 societies, 66 (48.5%) had an active presence on at least one SN. The most used SN was Facebook (n=60, 44.1%), followed by Twitter (n=37, 27.2%), YouTube (n=26, 19.1%), and Instagram (n=16, 11.8%). The SN with the largest number of followers was Facebook for 52 (78.8%) societies and Twitter for 12 (18.2%) societies. The number of followers was 361 (IQR 75-1806) on Twitter, 2494 (IQR 1049-5369) on Facebook, 1400 (IQR 303-3058) on Instagram, and 214 (IQR 33-955) on YouTube. There was a strong correlation between the number of posts and the number of followers on Twitter (*r*=0.95, 95% CI 0.91-0.97; *P*<.001), Instagram (*r*=0.83, 95% CI 0.58-0.94; *P*<.001), and YouTube (*r*=0.69, 95% CI 0.42-0.85; *P*<.001). According to the density of anesthetists in the country, there was no difference between societies with and without active SN accounts.

**Conclusions:**

Less than half of national anesthesia societies have at least one active account on SNs. Twitter and Facebook are the most used SNs.

## Introduction

In a globalized world, social networks (SNs) have taken a major place in the medical field and are essential tools for promoting research, medical innovations, and news from each specialty (eg, prompting novel techniques and disseminating new findings in congresses). For a medical society, the wide dissemination of each of its activities and news about its specialty is necessary to reach a large audience (eg, professionals of the sector, patients, residents, and medical students). In this context, the use of SNs by medical societies allows every information to be disseminated very quickly and at a low cost.

The most followed SNs are Facebook (2.79 billion users), YouTube (2.29 billion users), Instagram (1.29 billion users), and Twitter (396 million users) [1,2]. It has been recently described that among professionals working in anesthesia, intensive care medicine, and emergency medicine, 78% consulted Facebook, 41% Instagram, 40% YouTube, and 17% Twitter at least once a week [3]. This professional use of SNs is expected to increase with the arrival of young physicians, as it was reported that 35% of medical students used Twitter for teaching purposes [4]. Moreover, younger generations are increasingly using SNs as their primary means of finding information about a brand or society (this use even exceeds that of internet search engines among 16- to 24-year-olds), and the primary reason for using social media is to “stay up-to-date with news and current events” [1]. The time spent using SNs is constantly increasing from 1 hour and 51 minutes per day in 2015 to 2 hours and 25 minutes per day in 2020 [1]. Finally, several articles describe the value of using SNs (and in particular Twitter) for medical education [5,6]. Thus, more and more teachers and societies in several medical specialties are using SNs to highlight their educational content [7,8]. These elements illustrate the interest and importance for a society wishing to have visibility to position itself on SNs.

It is known that, for a given medical journal, articles that benefit from exposure on SNs are more cited than articles that are not [9,10]. Thus, more and more journals are using SNs to optimize their visibility into the scientific community. It is thus likely that a medical society present on SNs will be more visible to the medical community. The viral transmission of information that these networks allow is probably a key element to promote initiatives, valorize research results, and inform about trainings or congresses. However, although the presence of medical societies on SNs could be interesting for disseminating professional information, there is no study investigating their presence on SNs.

The objective of this study was to explore the worldwide presence and activity on SNs of national anesthesia societies.

## Methods

### Ethical Considerations

As a retrospective analysis of publicly available data that did not involve human subjects (and in accordance with French laws), this study was exempt from institutional ethics board review [11]. The results are reported in accordance with the Strengthening the Reporting of Observational studies in Epidemiology (STROBE) statement [12].

### Objectives

The main objective of this work was to describe the presence of the World Federation of Societies of Anesthesiologists (WFSA) member societies on the most popular SNs (ie, Twitter, Facebook, Instagram, and YouTube). The secondary objective was to assess the factors within societies (and their country) associated with the presence or absence of these societies from SNs.

### Data Collection

To limit the impact of profile variations on SNs, the entire data collection was carried out manually over 20 consecutive days in May 2021.

We determined the active presence or absence of WFSA anesthesia societies on SNs using the societies’ list available on the WFSA website [13]. An active presence on a given network was defined as the publication of at least one item of content (eg, post, tweet, and video) by the account over the 12 months preceding the collection. For each anesthesia society on each SN, the screening for finding SN accounts had a step-by-step procedure, which is as follows: (1) the name of the society was entered into the SN search engine using the language used to name the society on the WFSA website; (2) if no account was found after this first step, a similar search was carried out using the language of the country if it differed from the language used on the WFSA website (eg, Chinese and Arabic); (3) if no account was found after the second step, a similar search was conducted using the acronym of the society’s name (eg, “ASA” for the American Society of Anesthesiologists); and (4) if no account was found after the third step, we performed an internet search using the Google search engine and using the keywords [society name] and [SN name] (eg, “Taiwan Society of Anesthesiologists Twitter”).

If no account was found on an SN after the abovementioned steps, the society was considered not to have an active account on that network and was categorized as “absent.” An identified account of a society that had not published for more than a year was considered inactive and was therefore also categorized as “absent.”

When an account was found, the following data were collected from the public information presented on the accounts concerned: (1) for Twitter—number of tweets, number of followers, and year of creation of the account; (2) for Facebook—number of followers and year of creation of the account (there were no data concerning the number of posts for a given account on Facebook); (3) for Instagram—number of posts and number of followers (there were no data concerning the year of creation of a given account on Instagram); and (4) for YouTube—number of videos, number of followers, and year of creation of the account.

The following data for each society were collected from the WFSA website: preferred language of the society, number of society members, and number of physician anesthesia providers as well as their density in the country. For societies that did not indicate a preferred language on the WFSA website, the language that was considered to be preferred was that of the home country or the company’s website if it had one.

### Statistical Analysis

The values are presented as number and percentage (n, %) for qualitative variables, and as median (IQR) for quantitative variables. After ensuring the nonnormal distribution of the data by a Shapiro-Wilk test, quantitative variables were compared using a Mann-Whitney test. The qualitative variables were analyzed using a chi-square test. The Pearson correlation test was used to assess the strength of the association between 2 quantitative variables. A multivariable analysis was realized to identify factors related to anesthesia societies that were associated with the existence of at least one active account on SNs. Variables presenting *P*<0.3 in the univariable analysis were included in the multivariable analysis, which was performed using a logistic regression model with a backward stepwise model. The results are presented as odds ratio (OR) with 95% confidence intervals.

All statistical tests were 2-sided, and the .05 probability level was used to establish statistical significance. All statistics were produced using PRISM (v8.0.2, GraphPad Software) and MedCalc (v14, MedCalc Software Ltd) software.

## Results

### Population Description

**Figure 1:**
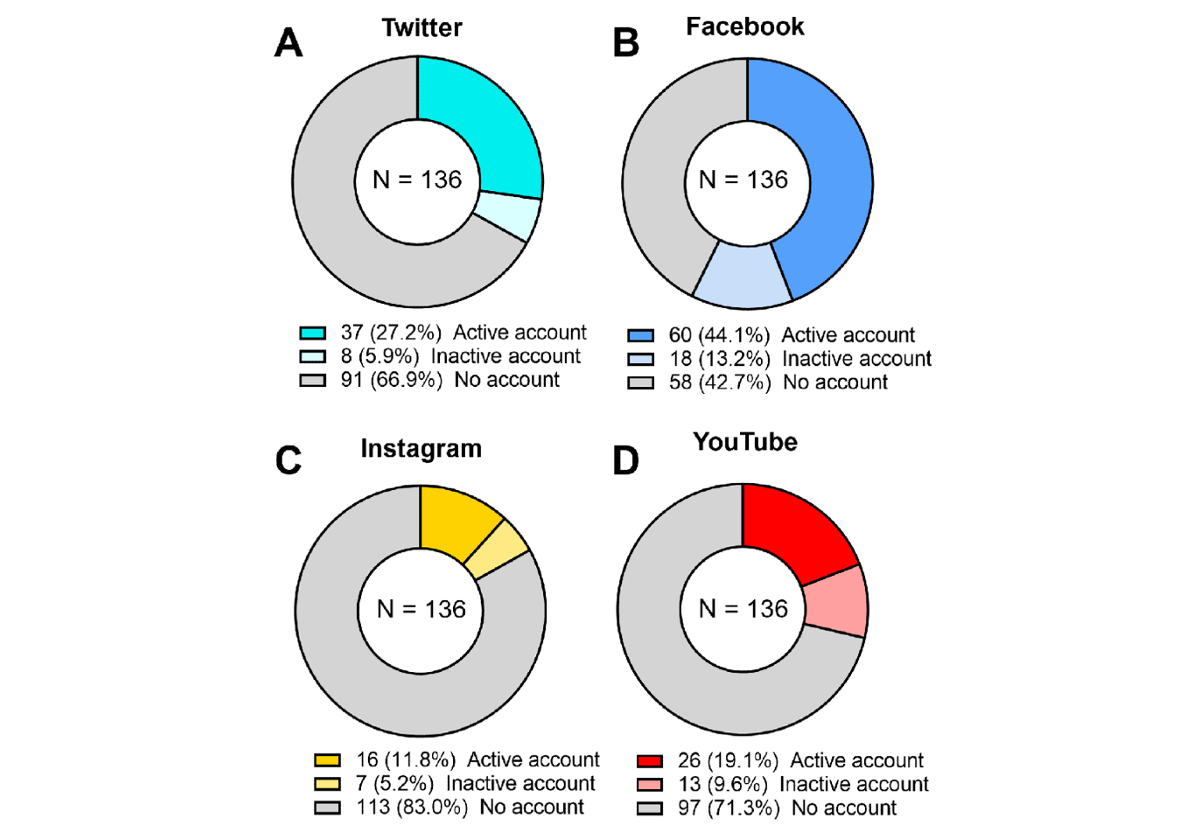
Proportion of national anesthesia societies with an account on Twitter (A), Facebook (B), Instagram (C), and YouTube (D). An active presence on a given network was defined as the publication of at least one item of content (eg, post, tweet, and video) by the account over the 12 months preceding the data collection; 136 societies were analyzed.

**Figure 2:**
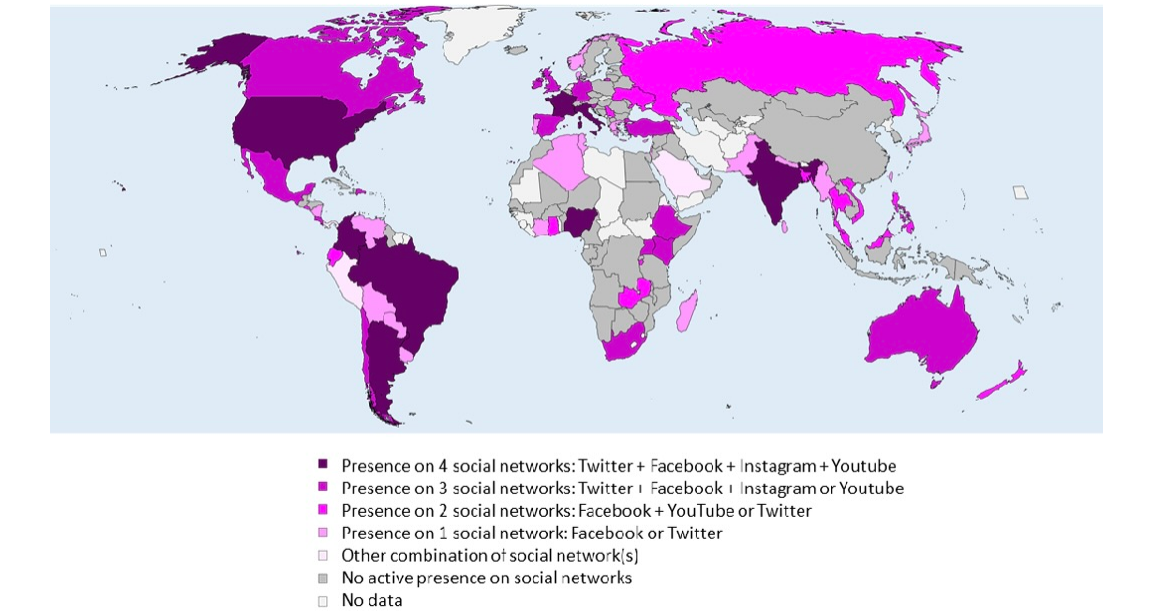
Number and type of social networks actively used by national anesthesia societies across the world.

**Figure 3:**
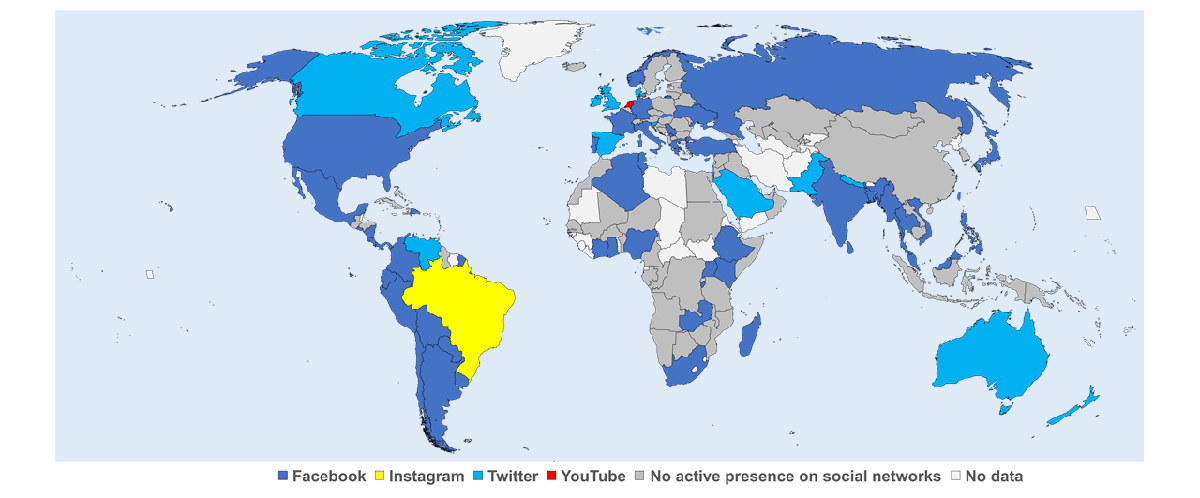
Active social network account with the largest number of followers among national anesthesia societies across the world.

A total of 136 anesthesia societies were analyzed. Of these 136 societies, 66 (48.5%) had an active presence on at least one SN. The most used SN was Facebook (60/136, 44.1%), followed by Twitter (37/136, 27.2%), YouTube (26/136, 19.1%), and Instagram (16/136, 11.8%). All SNs had a fraction of accessible but inactive accounts (Figure 1). The number, geographical location, and type of SNs actively used by national anesthesia societies are summarized in Figure 2 and supplemental Figure S1 (Multimedia Appendix 1). The SN with the largest number of followers was Facebook for 52/66 societies (78.8% of societies present on SNs) and Twitter for 12/66 societies (18.2%; Figure 3). Only 2 societies had Instagram (1/66 society; 1.5%) or YouTube (1/66 society; 1.5%) as their first source of followers (Figure 3).

### Activity of Anesthesia Societies on SNs

**Figure 4:**
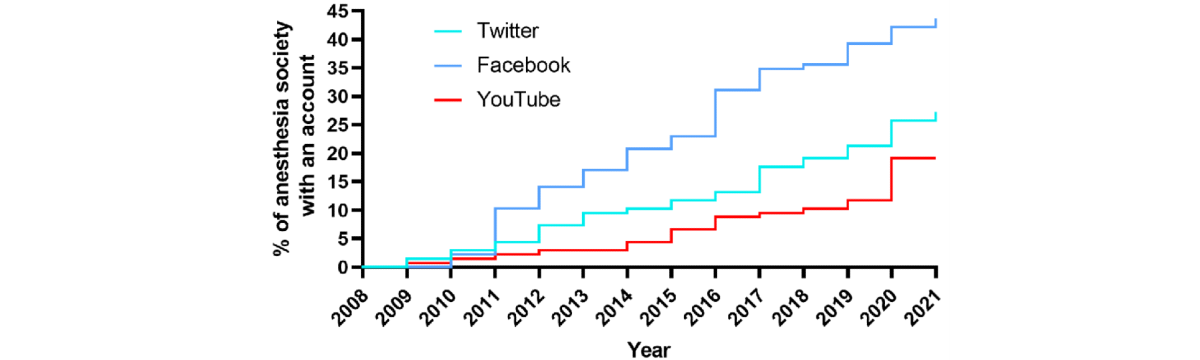
Proportion of national anesthesia societies with an active account on Twitter, Facebook, and YouTube over time. The ordinate scale is logarithmic. There were no publicly available data concerning the creation date of Instagram accounts.

The first anesthesia societies’ accounts on SNs were created in 2009-2010 with faster growth for the number of Facebook accounts; 2011 and 2016 were the 2 years with the highest number of account creation for this SN (>11 accounts per year; Figure 4). Growth in the number of accounts on other SNs was slower, with 2017 and 2020 being the years with the highest number of accounts created on Twitter (>6 accounts per year) and 2020 the year with the highest number of accounts created on YouTube (>10 accounts; Figure 4). There were no publicly available data concerning the creation date of Instagram accounts.

**Figure 5:**
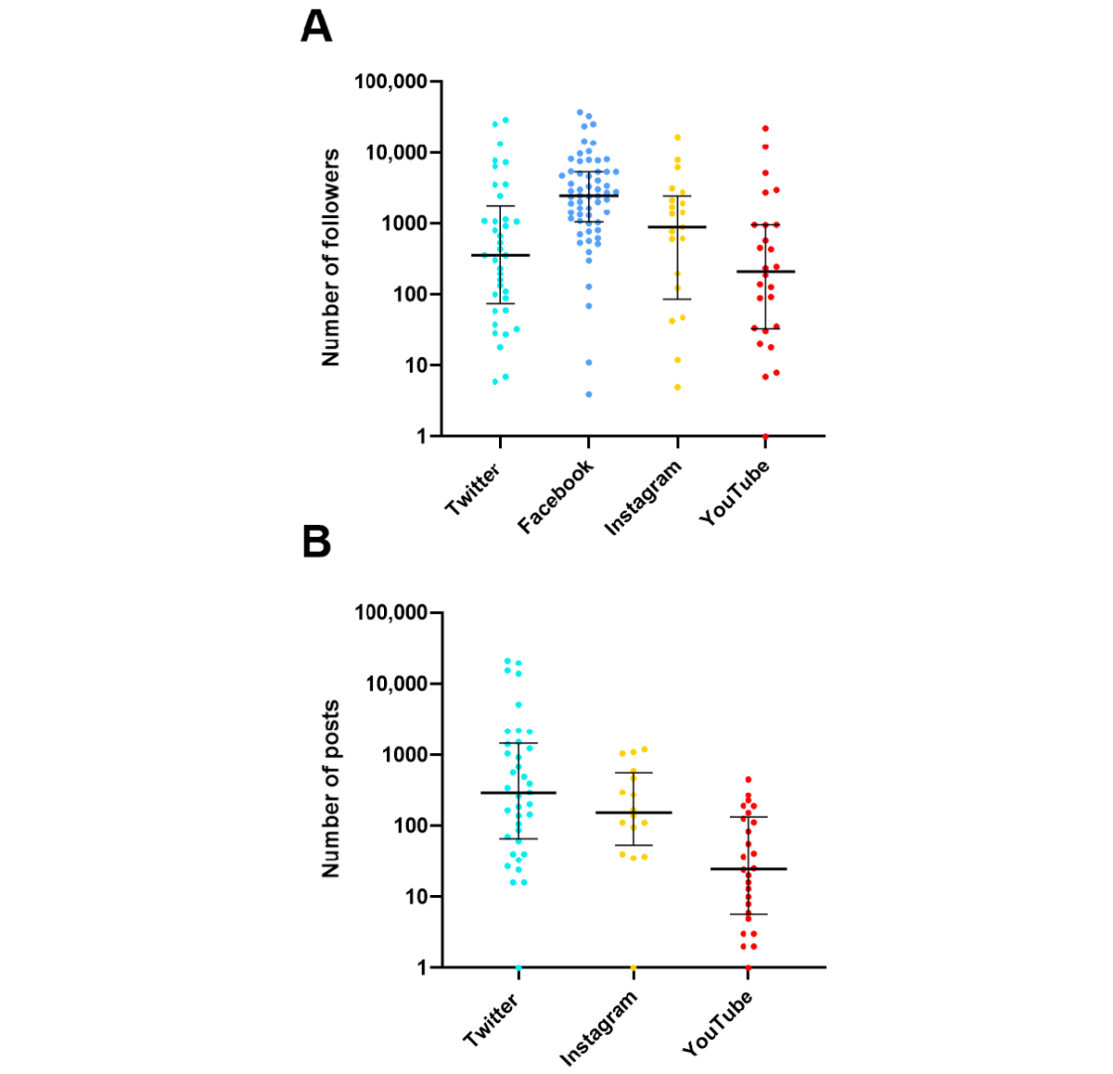
Numbers of followers (A) and posts (B) of national anesthesia societies' active social network accounts. The ordinate scale is logarithmic. The number of followers and posts are presented as dot plots with the median and interquartile range. There were no publicly available data on the number of Facebook posts over time.

Among the 66 societies with at least one active account on SNs, the number of followers was 361 (IQR 75-1806) on Twitter, 2494 (IQR 1049-5369) on Facebook, 1400 (IQR 303-3058) on Instagram, and 214 (IQR 33-955) on YouTube (Figure 5, part A). The number of posts (eg, tweet and video) on the accounts was 295 (IQR 66-1459) on Twitter, 152 (IQR 54-560) on Instagram, and 25 (IQR 6-132) on YouTube (there were no publicly available data on the number of Facebook posts; Figure 5, part B). The individual data for each company are available in Multimedia Appendix 2.

There was a strong correlation between the number of posts and the number of followers on Twitter (*r*=0.95, 95% CI 0.91-0.97; *P*<.001), Instagram (*r*=0.95, 95% CI 0.58-0.94; *P*<.001), and YouTube (*r*=0.69, 95% CI 0.42-0.85; *P*<.001). There was a correlation between the number of members of a society and the number of followers on Instagram (*r*=0.86, 95% CI 0.63-0.95; *P*<.001); however, there was no correlation between the number of members of a society and the number of followers on Twitter (*r*=–0.04, 95% CI –0.37 to –0.30; *P*=.82), Facebook (*r*=0.09, 95% CI –0.18 to 0.34; *P*=.52), and YouTube (*r*=0.20, 95% CI –0.21 to 0.55; *P*=.32). There was a moderate correlation between the number of physician anesthesia providers in the country and the number of followers on Twitter (*r*=0.55, 95% CI 0.27-0.75; *P*<.001) and Facebook (*r*=0.44, 95% CI 0.21-0.63; *P*=.001); however, there was no correlation between the number of physician anesthesia providers in the country and the number of followers on Instagram (*r*=0.49, 95% CI –0.01 to 0.79; *P*=.05) and YouTube (*r*=0.15, 95% CI –0.26 to 0.52; *P*=.46).

### Characteristics of National Anesthesia Societies With or Without Active Presence on SNs

Anesthesia societies with at least one active account on SNs had more members and were located in countries with a higher number of physician anesthesia providers (Table 1). There was no difference between societies with and without active social networking accounts according to the density of anesthetists in the country and the proportion of physician anesthesia provider members in the society (Table 1). The group of societies with at least one active account on SNs had more Spanish-speaking societies but fewer French-speaking societies compared with the group of societies without any account on SNs (Table 1).

We performed a multivariable analysis including the number of physician anesthesia providers, their density in the country, and the preferred language of the society. Spanish as society’s preferred language was associated with the existence of at least one active account on SNs (OR 5.39, 95% CI 1.46-20.00; *P*=.01). The number of physician anesthesia providers and their density in the country were not associated with the existence of at least one active account on SNs (OR 1.00, 95% CI 1.00-1.00; *P*=.25 and OR 1.02, 95% CI 0.97-1.06; *P*=.47, respectively).

**Table 1:** Characteristics of national anesthesia societies with or without an active presence on social networks.

Characteristics			With an active presence on at least one social network (n=66)		Without an active presence on social networks (n=70)		*P* value	
Anesthesia society members, n (IQR)			317 (100-1144)		70 (28-245)]		<.001	
Physician anesthesia providers in the country, n (IQR)			1050 (376-4464)		290 (60-1000)		<.001	
Physician anesthesia providers’ density in the country (per 100,000 population), n (IQR)			6.57 (1.67-15.17)		6.01 (0.62-12.5)		.22	
Percentage of physician anesthesia providers in the country, n (IQR)			44.8 (25.8-69.1)		42.8 (26.4-74.0)		.86	
**Preferred language of the society, n (%)**								.007
	English	42 (64)		52 (74)				
	Spanish	16 (24)		3 (4)				
	French	5 (8)		11 (16)				
	Other or unknown	3 (4)		4 (6)				

## Discussion

### Principal Findings

To our knowledge, we describe for the first time the presence and activity of national anesthesia societies on SNs. We also explore for the first time the link between these societies’ characteristics and their presence (or lack thereof) on SNs. Less than 50% of WFSA member societies have at least one active account on SNs. This number is low, especially since the first accounts were created more than 10 years ago. At a time when people spend almost 2.5 hours a day on SNs, it is interesting to note that many societies have not yet been willing or able to integrate SNs into their communication strategy [1]. However, it is interesting to note a recent (2020) increase in the number of anesthesia society accounts on Twitter and YouTube. This may reflect an awareness of the importance of having a wide visibility for a medical society, which is now partly achieved through these networks. The evolution of the number of societies present on SNs in the coming years will show whether this trend of increasing the number of accounts continues.

The worldwide distribution of societies with and without an active presence on SNs is heterogeneous. The majority of the active societies are from America, Western Europe, Southeast Asia, and Oceania. We could have made the hypothesis that low-income countries with less access to new information technologies would have less presence on SNs. Nevertheless, we observe that several anesthesia societies from high-income countries are also absent from SNs (eg, Sweden, Austria, Belgium, Switzerland, and South Korea) while several societies from transitional countries are active (eg, Burundi, Ghana, Madagascar, Venezuela, and Nigeria). Furthermore, the density of physician anesthesia providers was not associated with the presence or absence of a national society on SNs. These data suggest that, more than purely economic, demographic, or social factors, the presence or absence of a society on SNs may be the result of the individual initiatives of each society and its willingness to position itself or not on these networks. Thus, the fact that a Spanish-speaking society is more likely to have an active account on SNs compared with English- or French-speaking societies reflects the dynamics of South American societies on these networks. Finally, there are probably also geopolitical factors that may explain the absence of some societies (eg, Cuba and China) on SNs created by US companies. China also has its own ecosystem of SNs (eg, WeChat and Sina-Weibo), and it is therefore likely that the communication of the Chinese Society of Anesthesiology (which represents 72,000 physician anesthesia providers) is carried out through these national networks.

By far, the most used SN by anesthesia societies is Facebook, which has more and older accounts. Facebook is also the SN with the highest number of followers for the majority of societies. This is consistent with the fact that it is the SN with the most users in the world [1]. If Facebook is a network used by all age groups (in 2020, 76% of 18- to 24-year-olds used it vs 79% of 30- to 49-year-olds), Instagram and Twitter are networks used mainly by young people with respectively 75% and 44% of users among 18- to 24-year-olds (vs 47% and 26% among 30- to 49-year-olds, respectively) [14]. Moreover, while 61% of Facebook users have attended university, 69% of Instagram users and 75% of Twitter users did the same [15]. Finally, while men constitute the majority of Facebook and Twitter users (56% and 68% of users, respectively), women represent the majority on Instagram (57%) [16]. Therefore, Instagram and Twitter networks reach, on average, younger people with a higher level of education compared with Facebook; and more women use Instagram compared with any other SN platform. Taken together, these data suggest that it appears important to increase the presence of anesthesia societies on Twitter and Instagram to gain and maintain visibility among the younger educated generations, part of which comprises junior physicians and medical students. In this context, it is interesting to note that several large anesthesia societies with a strong presence on SNs have the most followers on Twitter (eg, Australia, New Zealand, Canada, Spain, United Kingdom, and Ireland).

Regardless of the SNs analyzed, the number of followers was strongly correlated to the volume of publication of the account. Maintaining and improving one’s presence on SNs therefore requires regular publication of information about the society and the specialty, which is a constant task requiring a real investment of time and sometimes money from the society. This perhaps explains why, on all SNs, there was a variable proportion of accounts created, which were inactive due to a lack of organized logistics or lack of willingness to maintain the accounts. The number of followers on Facebook and Twitter correlated with the number of physician anesthesia providers in a given country but not with the number of society members in that country. These data may suggest that Facebook and Twitter accounts reach a broad audience (ie, all physician anesthesia providers) beyond the society members who originate the account. The fact that the number of followers on Instagram correlates strongly with the number of members of the society but not with the overall number of physician anesthesia providers suggests, on the other hand, that the followers of this SN mainly come from the society itself and that it reaches less broadly the whole anesthetic community in a country. However, these suggestions are only hypotheses, and more detailed surveys within each country would be needed to interpret these results with certainty.

### Limitations

Despite the interesting results, our study has several limitations. First, this work was limited to anesthesia societies that are members of the WFSA. The selection of this population allowed us to have access to information on the societies available on the WFSA website (eg, preferred language and number of members) and enabled us to analyze only the existing and active societies. However, we did not include all national societies worldwide. The fact that the WFSA includes a majority of the existing societies probably limits the bias induced by this choice. Second, the data on the number of physician anesthesia providers in a country and their density are from the 2015-2016 period [17]. New measurements are currently underway, but it is possible that some of these data have changed between 2015 and 2021. However, it is unlikely that there has been a major change in density or medical ratios during this period. Third, even a standardized manual account search procedure has its flaws. Some societies may use a pseudonym other than their society name or an acronym or misspelled name when they register. It is therefore possible that our referencing, while rigorous, has missed some societies active on SNs. Fourth, we only focused on 4 SNs. However, since Facebook, Twitter, Instagram, and YouTube are part of the 5 most visited website in the world and given the low rate of the anesthesia societies’ presence on these major networks, it would probably have been wasteful to seek their presence on other SNs [1]. Fifth, we did not assess the relative impact of social media presence for the societies studied (eg, likes or retweets per post, number of visualizations of videos, and number of followers). We can thus describe the presence of these societies on social media but cannot define the impact of such presence on their visibility.

In conclusion, the rate of national anesthesia societies having at least one account on SNs appears relatively low in view of societal developments in SN use. This low presence rate suggests that there is still significant room for improvement in highlighting anesthesia on SNs. Each medical society could consider its communication strategy and give itself the means to use this communication tool to promote its activity and initiatives. The active or inactive presence of a society on SNs does not seem to be influenced by the socioeconomic context or the density of anesthetists in the country. Thus, being present on SNs appears to be more the result of a strategic choice by the society than the human or material means available to achieve this.

### What is Known

- SNs have taken a major place in the medical field, but the worldwide presence of national anesthesia societies on SNs is not known.

### What is New

- Among 136 societies, 66 (48.5%) had presence on at least one SN.
- The most used SN was Facebook (60/136, 44.1%), followed by Twitter (37/136, 27.2%).
- Less than half of anesthesia societies have an active account on SNs.

## Appendix

Multimedia Appendix 1 Presence on social networks of national anesthesia societies that are members of the World Federation of Societies of Anesthesiologists. Each line corresponds to a country, and each column to a social network. If the national society is present on a given network, the corresponding box is colored (light blue for Twitter, dark blue for Facebook, yellow for Instagram, and red for YouTube). If the society is not present on the social network, the box is gray.

Multimedia Appendix 2 Individual data of social network activity for each anesthesia society.

## Abbreviations

OR: odds ratio
SN: social network
STROBE: Strengthening the Reporting of Observational studies in Epidemiology
WFSA: World Federation of Societies of Anesthesiologists

## Data Availability

The raw data supporting the conclusions of this manuscript can be made available upon request by the authors to any qualified researcher.
