# Supplementary material for: Worldwide Presence of National Anesthesia Societies on Four Major Social Networks in 2021: Observational Case Study
Source: JMIR Perioper Med. 2022 Jul 20;5(1):e34549. doi: 10.2196/34549 (PMC9350816; doi:10.2196/34549)
Supplement: Multimedia Appendix 1 [file periop_v5i1e34549_app1.pdf]

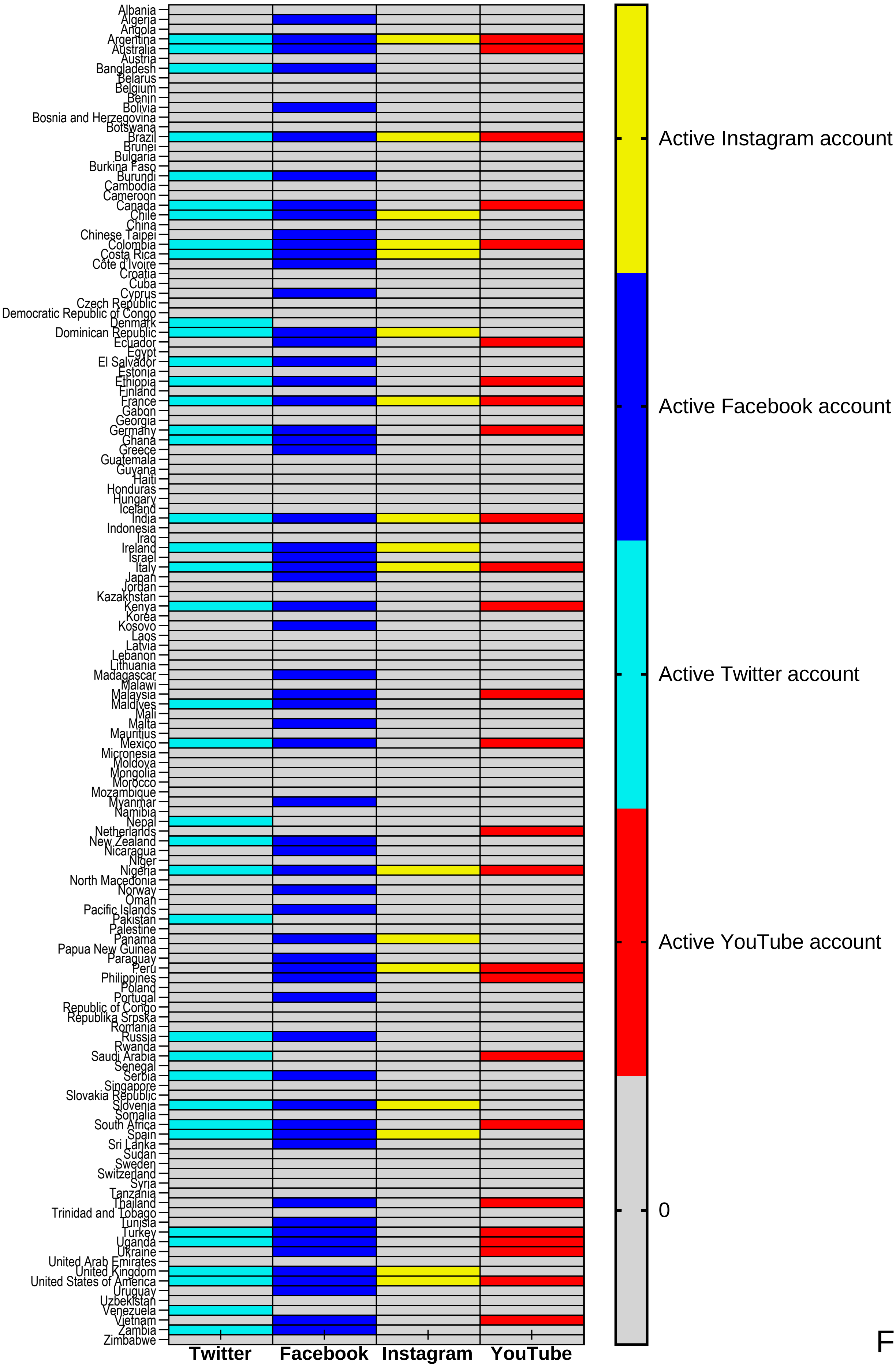
Albania
Algeria
Angola
Argentina
Australia
Austria
Bangladesh
Belarus
Belgium
Benin
Bolivia
Bosnia and Herzegovina
Botswana
Brazil
Brunei
Bulgaria
Burkina Faso
Burundi
Cambodia
Cameroon
Canada
Chile
China
Chinese Taipei
Colombia
Costa Rica
Côte d'Ivoire
Croatia
Cuba
Cyprus
Czech Republic
Democratic Republic of Congo
Denmark
Dominican Republic
Ecuador
Egypt
El Salvador
Estonia
Ethiopia
Finland
France
Gabon
Georgia
Germany
Ghana
Greece
Guatemala
Guyana
Haiti
Honduras
Hungary
Iceland
India
Indonesia
Iraq
Ireland
Israel
Italy
Japan
Jordan
Kazakhstan
Kenya
Korea
Kosovo
Laos
Latvia
Lebanon
Lithuania
Madagascar
Malawi
Malaysia
Maldives
Mali
Malta
Mauritius
Mexico
Micronesia
Moldova
Mongolia
Morocco
Mozambique
Myanmar
Namibia
Nepal
Netherlands
New Zealand
Nicaragua
Niger
Nigeria
North Macedonia
Norway
Oman
Pacific Islands
Pakistan
Palestine
Panama
Papua New Guinea
Paraguay
Peru
Philippines
Poland
Portugal
Republic of Congo
Republika Srpska
Romania
Russia
Rwanda
Saudi Arabia
Senegal
Serbia
Singapore
Slovakia Republic
Slovenia
Somalia
South Africa
Spain
Sri Lanka
Sudan
Sweden
Switzerland
Syria
Tanzania
Thailand
Trinidad and Tobago
Tunisia
Turkey
Uganda
Ukraine
United Arab Emirates
United Kingdom
United States of America
Uruguay
Uzbekistan
Venezuela
Vietnam
Zambia
Zimbabwe
Twitter
Facebook
Instagram
YouTube
Active Instagram account
Active Facebook account
Active Twitter account
Active YouTube account
0

Figure S1

**Supplemental Figure S1. Presence on social networks of national anaesthesia societies which are members of the World Federation of Societies of Anesthesiologists.** Each line corresponds to a country and each column to a social network. If the national society is present on a given network, the corresponding box is colored (light blue for Twitter, dark blue for Facebook, yellow for Instagram and red for YouTube). If the society is not present on the social network, the box is grey.
